# Negative and neutral results matter in abdominal wall surgery

**DOI:** 10.3389/jaws.2026.17443

**Published:** 2026-08-20

**Authors:** Manuel López-Cano, Josep M. Garcia-Alamino

**Affiliations:** 1 Abdominal Wall Surgery Unit, Department of General Surgery, Hospital Universitari Vall d’Hebron, Universitat Autònoma de Barcelona, Barcelona, Spain; 2 School of Health Sciences, Blanquerna Ramon Llull University, Barcelona, Spain

**Keywords:** abdominal wall surgery, evidence-based surgery, hernia, negative results, neutral results

## Introduction

Scientific progress depends on identifying interventions that work but also on recognising those that fail to provide the expected benefit those that perform similarly to existing alternatives and those for which uncertainty remains.

Publication bias has traditionally been defined as the preferential submission or publication of studies reporting positive or statistically significant findings [1]. Favourable trials are more likely to be published than studies with negative, neutral or inconclusive findings [2]. Distortion can occur through the non-publication of entire studies or through selective reporting of favourable outcomes within published manuscripts [3].

Positive findings exceed negative results in the general and orthopaedic surgical literature raising concerns about the comprehensiveness of the evidence available to clinicians [4].

Abdominal wall surgery is especially at risk. The discipline is characterised by rapid technical innovation, diversity of procedures and continuous introduction of new meshes, fixation systems, minimally invasive approaches and robotic platforms. Early favourable studies often attract attention. Studies showing no superiority, no clinically meaningful difference or an unfavourable balance between benefits and harms may receive less visibility.

## Defining negative, neutral and inconclusive results

The terms negative, neutral, null and non-significant are often used interchangeably although they describe different situations.

A negative result generally indicates that the findings do not support the original hypothesis or suggest that the intervention performs worse than expected. A neutral or null result usually means that no statistically significant difference was detected between the study groups. An inconclusive result indicates that the available evidence is insufficiently precise to determine whether a clinically important difference exists.

None of these findings automatically demonstrates equivalence.

A superiority trial that fails to reject the null hypothesis does not prove that two treatments are the same. Absence of statistical significance may reflect a genuinely absent or small treatment effect but it may also result from inadequate sample size, low event rates, imprecise measurement, incomplete adherence or insufficient follow-up. Abdominal wall surgery studies are particularly vulnerable to type II error (i.e., false negative) because sometimes recruitment is difficult and relevant outcomes such as recurrence may require prolonged observation [5].

Equivalence and non-inferiority require dedicated designs and appropriate statistical analyses. Authors should therefore avoid claiming that treatments are equivalent merely because a conventional superiority comparison produces a P value greater than 0.05 (i.e., not statistically significant).

This distinction is essential when advocating publication of neutral research. The aim is not to increase the volume of uninterpretable non-significant findings. It is to ensure that well-designed studies are evaluated according to the relevance of their question and methodological quality rather than according to whether they produce a positive result.

## Why neutral results are clinically valuable

A reliable finding that two procedures achieve similar recurrence rates may be highly informative when one option is less costly, simpler to perform or associated with shorter operative time. Comparable outcomes may support a less invasive approach, challenge unnecessary technological investment or justify the omission of an operative step.

Neutral studies also define the limits of innovation. A new procedure does not need to be harmful to lack clinical value. It may simply fail to improve outcomes sufficiently to compensate for greater complexity, cost, resource use or learning requirements.

In comparative effectiveness research demonstrating no clinically meaningful advantage may be precisely the information needed for rational decision-making. Surgeons and patients do not only need to know whether a difference is statistically detectable. They need to know whether its magnitude is large enough to matter and to justify additional risks or costs.

Negative and neutral findings therefore serve complementary purposes. Both can prevent premature adoption, reduce unnecessary costs and redirect future research.

## Publication bias is a collective responsibility

Publication bias develops through multiple decisions involving researchers, sponsors, conference committees, reviewers and journals.

Researchers may decide not to submit manuscripts when results fail to confirm the original hypothesis or may also delay submission or abandon a study because they assume that editors will consider it uninteresting [6, 7].

Editorial preferences may reinforce this imbalance when novelty or citation potential are prioritised over reproducibility and clinical utility.

Publication bias should therefore be regarded as a cultural and systemic phenomenon. Academic advancement rewards outstanding results. Industry sponsors may favour evidence of benefit. Conferences seek engaging presentations, journals compete for readers' attention and readers are naturally drawn to apparent breakthroughs. Under these conditions studies showing similarity, uncertainty or failure to improve outcomes may be perceived as unsuccessful even when methodologically sound.

Correcting the imbalance requires shared responsibility.

## Why abdominal wall surgery needs the entire evidence base

Abdominal wall surgery combines substantial heterogeneity in patient characteristics, defect complexity, mesh properties, mesh position, fixation, fascial closure and surgical expertise. Outcomes are also affected by learning curves and duration of follow-up. The rapid adoption of surgical technologies creates additional vulnerability. New approaches are often introduced through feasibility studies, institutional series or reports from expert centres. Such studies are valuable during technical development but may not predict comparative effectiveness in routine clinical practice.

The comparison between robotic and laparoscopic ventral hernia repair illustrates the importance of publishing neutral findings. In a multicentre randomised trial robotic repair did not reduce hospital stay during the first 90 postoperative days while requiring longer operating times and incurring higher costs [8]. Subsequent evaluation of patient-reported outcomes found broadly comparable results between the two approaches [9].

These studies do not imply that robotic surgery lacks value across all clinical settings nor do they establish universal equivalence between the two techniques. Rather they demonstrate that the anticipated advantages of a more technologically advanced platform were not evident for the outcomes evaluated. Such evidence is essential for informing clinical decision-making, guiding resource allocation and ensuring that the adoption of new technologies is supported by robust comparative evidence.

Mesh fixation provides another example. A randomised trial of laparoscopic inguinal hernia repair found no early recurrence benefit from fixation while fixation increased costs and was associated with more chronic pain [10]. A systematic review of randomised trials in open inguinal repair similarly found no convincing differences in recurrence or infection between fixation strategies [11].

In these cases neutral recurrence findings were not an absence of evidence. They helped determine whether an additional technical step produced sufficient value. When effectiveness is comparable differences in pain, cost, operative time and technical complexity become more important.

Prophylactic mesh for parastomal hernia prevention offers an example of evidence challenging initial optimism. The STOMAMESH randomised trial found that sublay mesh did not prevent parastomal hernia [12]. Five-year follow-up from the PREVENT trial reported that prophylactic mesh was safe but did not reduce long-term incidence [13]. A pooled long-term analysis of cohorts from three randomised trials likewise concluded that prophylactic mesh did not prevent parastomal hernia over extended follow-up [14]. A recent systematic review and meta-analysis of eight randomized trials with long-term follow-up appear to cast doubt on the initial optimism [15]. However, these findings should not be interpreted as proving that every prophylactic mesh strategy is ineffective. Mesh configuration, position, operative technique, patient selection and outcome definition remain relevant and still unresolved from a data perspective. Their importance lies in preventing a simplistic narrative of universal benefit and in identifying the questions that remain open.

Other studies show why the positive-negative dichotomy could be inadequate. Prophylactic intraperitoneal mesh may reduce incisional hernia while increasing early pain or wound-related morbidity [16]. Lightweight mesh may reduce chronic discomfort while raising concerns about recurrence in selected settings [17]. These studies reveal trade-offs rather than purely positive or negative outcomes.

## Some consequences of selective publication

Selective publication alters the apparent balance between benefits and harms. When positive findings are visible but neutral or unfavourable results remain unavailable meta-analyses may overestimate treatment effects and underestimate uncertainty.

Systematic reviews cannot incorporate studies that cannot be located. Guideline panels cannot assess unpublished clinical data, failed replications or long-term follow-up. Recommendations may consequently appear stronger and more definitive than the total evidence justifies.

Non-publication also creates research waste. Clinical studies require protocol development, ethical review, recruitment, data collection, follow-up and analysis. When valid findings remain unpublished because they are considered insufficiently exciting much of their scientific and ethical value is lost [18].

It also permits unnecessary actions. Other groups may inadvertently repeat interventions that have already failed to demonstrate benefit. Additional patients may then be exposed to interventions that previous research had already shown to be ineffective, non-superior, or unlikely to warrant further research simply because those findings were not made publicly available. Even an inconclusive study can inform sample-size calculations, feasibility, outcome selection and future trial design, provided its uncertainty and limitations are reported transparently. Beyond its scientific consequences, non-publication also raises ethical concerns. Participants contribute to research with the expectation that their involvement will generate knowledge to benefit future patients failure to make study findings publicly available undermines that social and ethical commitment.

## Moving beyond statistical significance

The conventional classification of studies according to whether P is below 0.05 has contributed to the marginalisation of neutral findings. Statistical significance does not measure clinical importance and a non-significant result does not establish absence of effect.

A small statistically significant difference may be clinically trivial. Conversely a non-significant estimate with a sufficiently narrow confidence interval may exclude any benefit large enough to justify a more costly or complex intervention.

Interpretation should therefore focus on effect size, confidence intervals, clinically meaningful thresholds, complications, patient-reported outcomes, resource use and long-term durability. Authors should describe the range of effects compatible with the data rather than simply stating that there was “no difference”.

The EQUATOR (Enhancing the QUAlity and Transparency Of Health Research) Network promotes the use of reporting guidelines to ensure transparent and complete reporting of health research [19]. Adherence to these guidelines is particularly important for studies with neutral findings as it enables readers to assess whether the results provide credible evidence.

## Priorities for change

Researchers should register comparative studies and define methodology before results are known. Completed studies should be submitted for publication regardless of whether the original hypothesis was supported.

Authors should avoid describing non-significant findings as evidence of equivalence without considering statistical power and confidence intervals. Equivalence and non-inferiority should only be claimed when supported by an appropriate design.

Reviewers should evaluate the relevance of the clinical question, risk of bias, precision and transparency before considering the direction of the findings.

Editors should explicitly welcome negative, neutral, replication and long-term follow-up studies. Editorial decisions should be based on methodological validity rather than on statistical significance or perceived novelty.

Scientific societies should provide non-positive research with appropriate visibility at conferences and include it in consensus processes and educational activities. Funding bodies should require dissemination plans that apply independently of study outcome.

Systematic reviewers and guideline developers should search trial registries, protocols and conference proceedings for completed but unpublished studies. When selective publication is suspected confidence in the evidence should be reduced.

## Discussion

Negative and neutral studies are essential components of surgical evidence. Nevertheless, these categories should be interpreted with caution. Failure to demonstrate superiority should not be construed as evidence of equivalence, particularly when statistical power is insufficient. Studies with inadequate sample sizes or methodological limitations do not become informative simply because they yield non-significant results. Regardless of the direction of the findings methodological and reporting standards must be applied [19].

The solution to publication bias is not the unselective publication of every study reporting null findings but the dissemination of clinically relevant, methodologically rigorous and transparently reported research.

Abdominal wall surgery requires a comprehensive, long-term assessment of multiple clinically relevant outcomes. Recurrence, postoperative pain, wound morbidity, mesh-related complications, quality of life, operative efficiency and costs may evolve differently over time. Consequently an intervention may demonstrate benefit for one outcome while showing little or no advantage for others. Reducing such evidence to a simple dichotomy of positive or negative findings oversimplifies the complexity of abdominal wall surgery and may obscure information essential for informed, shared decision-making in the context of the inevitable “surgical ignorance” that characterizes clinical decision-making [20, 21].

A specialty that publishes only positive findings cannot reliably assess the true value of its interventions.
